# Cloning, Expression and Purification of Subunit H of Vacuolar H^+^-ATPase from *Mythimna separata* Walker (Lepidoptera: Noctuidae)

**DOI:** 10.3390/ijms150915443

**Published:** 2014-09-01

**Authors:** Lina Lu, Zhijun Qi, Wenjun Wu

**Affiliations:** Institute of Pesticide Science, Northwest Agriculture and Forestry University, Yangling 712100, Shaanxi, China; E-Mails: chinalinalu@gmail.com (L.L.); qzhij@163.com (Z.Q.)

**Keywords:** vacuolar (H^+^)-ATPases, *Mythimna separata*, subunit H, insect expression system

## Abstract

The vacuolar (H^+^)-ATPase (V-ATPase) of insect, which is composed of membrane-bound V_0_ complex and peripheral V_1_ complex, participates in lots of important physiological process. Subunit H, as a subunit of V_1_ complex, plays a vital role in bridging the communication between V_1_ and V_0_ complexes and interaction with other proteins. Yeast subunit H has been successfully crystallized through expression in *E. coli*, but little is known about the structure of insect subunit H. In this study, we cloned, expressed and purified the subunit H from midgut of *Mythimna separata* Walker. Through RACE (rapidly amplification of cDNA ends) technique, we got 1807 bp full length of subunit H, and to keep the nature structure of subunit H, we constructed Baculovirus expression vector with His-tag in the *C*-terminal and expressed the recombinant protein in insect sf9 cells, thereafter, purified the recombinant protein by Ni-NTA columns. Results of SDS-PAGE, western blotting and mass spectrometry showed that the recombinant protein was successfully expressed. The method of expressing and purifying *M. separata* subunit H will provide a foundation for obtaining the crystal of subunit H and further study of the design of novel insecticides based on its structure and function.

## 1. Introduction

The vacuolar H^+^-ATPase (V-ATPase) is one of the fundamental enzymes in organisms. Some of its subunits may have the same ancestor as F-ATPase [1]. Though their structure and action mechanism are similar, their functions are completely different from each other. Unlike F-ATPase which produces ATP to provide energy for eukaryotic cells, the V-ATPase consumes ATP to transport H^+^ and provides force for many other secondary transport processes [1,2].

V-ATPase is mainly located at endomembranes and plasma membranes [3]. In endomembranes, V-ATPase acidifies specific organelles such as endosomes, lysosomes, and secretory vesicles in every eukaryotic cell [4]; while in plasma membranes of many animal cell types, they are involved in pH homeostasis and membrane energization [5].

For insects, besides the function of acidifying specific intracellular organelles, V-ATPase also plays a vital role in transepithelial cation transport in epithelia such as salivary glands, labial glands, midgut and sensory sensilla, by cooperating with K^+^/H^+^ antiporter and ion channels. The V-ATPase from goblet cell apical membranes of the tobacco hornworm midgut is the first insect vacuolar-type ATPase found in plasma membrane, and it is responsible for the alkalinization of the gut lumen [5,6]. Like other organisms, the insect V-ATPase is also a multisubunit enzyme, which is composed of V_1_ complex and V_0_ complex. The peripheral V_1_ complex contains eight different subunits (A, B, C, D, E, F, G and H), and the membrane bound V_0_ complex includes ac_n_de subunits with the possible c isoforms c' and c'' [7,8]. The results of studies on the V-ATPase from yeast and clathrin-coated vesicles show that subunit H is localized at the base of V_1_ complex near the interface between the V_1_ and V_0_ domains. Furthermore, subunit H in yeast has been shown to play an essential role in silencing ATP hydrolysis by free V_1_ through bridging the rotary and stator domains [7]. However, little is known about the detailed structure and function of insect subunit H.

The yeast subunit H expressed in *E. coli* has been purified and crystallized [9], but the expression, crystallizing and structure of insect H subunit have not been reported so far. It is undoubted that H subunit in yeast is different from in insect. To further understand the structure and characters of H subunit in lepidoptera insect, especially to probe the possibility that the H subunit of insect could be exploited as an insecticidal target, we cloned, expressed and purified the subunit H of *M. separata* Walker in the insect expression system, which is better than the prokaryotic expression system. Additionally, the modification process, such as posttranslational processing, will bring the structure of recombinant protein closer to the natural structure. The method and purification of recombinant protein could provide basis for further study of getting a large amount of subunit H to analyze the crystal structure and function.

## 2. Results

### 2.1. Cloning and Identification of PCR and RACE Products

**Figure 1:**
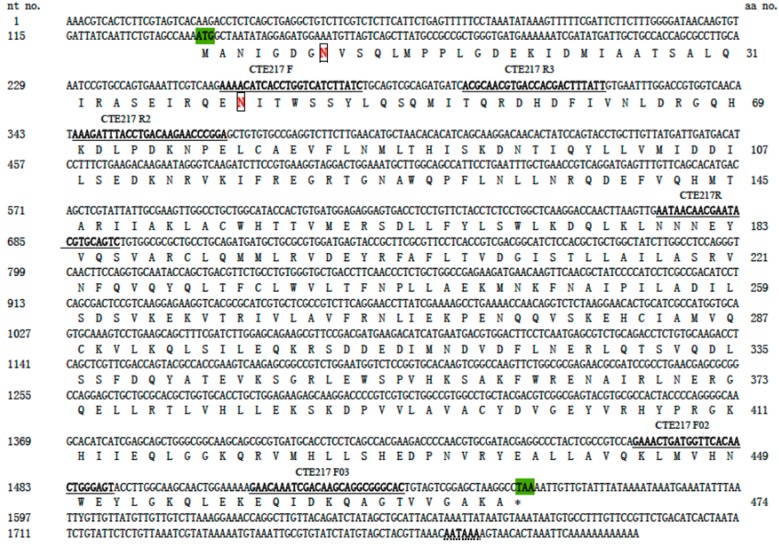
The full-length cDNA sequence of *MsV1H* and deduced amino acid sequence. The start and stop codon are in green, and putative polyadenylation signal sequences are indicated by a dotted line. Primers were labeled and unlined. Two potential Asn-linked glycosylation sites were boxed.

By using cDNA from the midgut of *M. separata* as template and primers showed in Table 1, the expected *ca.* 439 bp PCR product was obtained. After sequencing the PCR product, specific primers (Table 1) for RACE were designed; we took 1058 bp 3' RACE and 235 bp 5' RACE products and sequenced them. PCR products and the 5' and 3' RACE products were aligned to form a contig to get the full length. The 3' end contains polyadenylation signals typical of eukaryotes. Therefore, the sequence appears to contain the complete 3' ends. The cDNA encoding V-ATPase of *M. separata* was named as *MsV1H*, which was then deposited in GenBank with the accession number KC683729. The full length of *MsV1H* is 1807 bp with an open reading frame (ORF) of 1425 base pairs, a 5'-untranslated region (UTR) of 136 bp, and a 3'-UTR of 246 bp (Figure 1). And the ORF of *MsV1H* encoded a polypeptide of 474 amino acids with a calculated molecular weight of about 54.8 kDa and an isoelectric point (pI) of 6.26.

**Table 1:** Primers used in PCR amplification and RACE reaction.

Name	Sequence (5'-3')	Primer Used
CTE217 F	AAAACATCACCTGGTCATCTTATC	RT-PCR
CTE217 R	GACTGCACGTATTCGTTGTTATT	RT-PCR
CTE217 F02	GAAACTGATGGTTCACAACTGGGAGT	3' RACE
CTE217 F03	GAACAAATCGACAAGCAGGCGGGCAC	3' RACE
CTE217 R2	TCCGGGTTCTTGTCAGGTAAATCTTT	5' RACE
CTE217 R3	AATAAAGTCGTGGTCACGTTGCGT	5' RACE
QHF	GCCGCTGGGTGATGAAA	qPCR
QHR	GTGTTGTCCTTGCTGATGTGTG	qPCR
QActinF	GGTGTGATGGTTGGTATGGGT	qPCR
QActinR	TCGTTGTAGAAGGTGTGGTGC	qPCR

Searching the PROSITE database revealed that there are no conservative motifs. By using the NCBI BLASTp, the result shows the deduced amino acid belongs to V-ATPase subunit H superfamily. The *N*-terminal (28–328 residues) and *C*-terminal (333–451 residues) domain belongs to the V-ATPase-*H*-*N* superfamily and V-ATPase-*H*-*C* superfamily, respectively.

### 2.2. Phylogenetic Analysis

**Figure 2:**
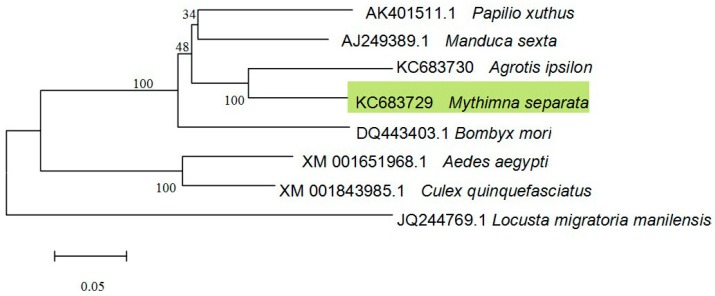
Phylogenetic relationship of V-ATPase subunit H. This un-rooted phylogenetic tree was constructed by the neighbor-joining method. Nodes indicate bootstrap calculated with 1000 replications support.

A phylogenetic tree was constructed using the neighbor-joining method (Figure 2) to show the conservative relationship of *MsV1H* with subunit H of other insects. The analysis shows the *MsV1H* is located on the same branch with *Agrotis ipsilon* Rott [10] and both of them are located on the same branch with *M. sexta* and *Papilio xuthus* with 48% bootstrap support, indicating *MsV1H* shared the closest relationship with subunit H of *M. sexta* and *P. xuthus*. Furthermore, *MsV1H*, subunit H of *A. ipsilon*, *M. sexta* and *P. xuthus* are all on the same branch with *B. mori*, which is consistent with the result of amino acid alignment.

Subunit H of *M. separata* is one of the subunits of V_1_ complex, which is located in cytoplasm, so it should be hydrophilic, which matches the result computed by ProtParam tool (http://web.expasy.org/protparam/). Although the MsV1H has no signal peptide, it has two potential Asn-linked glycosylation sites predicted by NetNGlyc 1.0. (CBS, Lyngby, Denmark).

### 2.3. Developmental Expression Profiles

**Figure 3:**
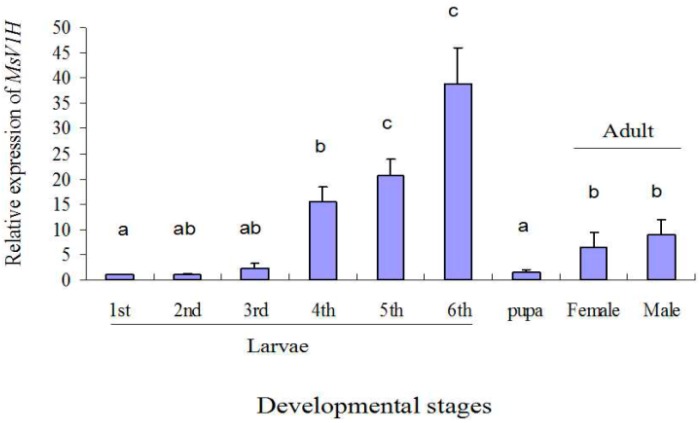
Relative expression level of *M. separata* *MsV1H* in different developmental stages. Expression level was normalized using β-actin as the standard. The normalized value was applied to relative expression analysis. The results are shown as the mean ± SE. The error bars show the ranges of standard errors. Letters on the error bars indicate the significant differences by ANOVA analysis (*p* < 0.05).

To determine the relative expression patterns of *MsV1H* mRNAs at the various developmental stages, a RT-qPCR was conducted. The *MsV1H* transcript was detectable from larvae to adult, and was significantly higher in the sixth instar than at other stages (Figure 3). The results showed that no significant difference in *MsV1H* expression was observed from first to third instar. There was a rapid increase of *MsV1H* expression at the fourth instar of *M. separata*, and it reached a peak in the sixth instar larvae (38.90-fold of the first instar). The gene expression level dropped at the pupal stage (1.57-fold of the first instar) and finally increased in adults (6.41- and 8.98-fold in female and male, respectively).

### 2.4. Expression, Purification and Identification of Subunit H from M. separata

Recombinant protein was purified by Ni-NTA column after Sf9 cells were transfected with either the control or the recombinant Baculovirus with coding sequence of *MsV1H*. Flow-through elution of each part was shown in Figure 3. Purified MsV1H protein was eluted twice by 500 mM imidazole.

**Figure 4:**
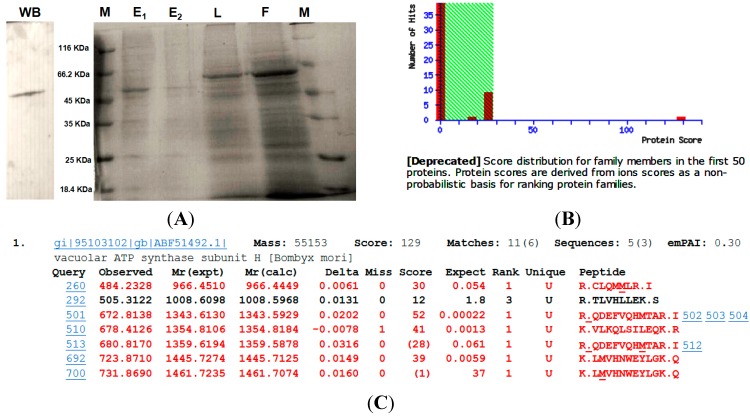
(**A**) Purification of MsV1H and identification by western blotting. WB: western blotting; M: protein marker; E_1_: 1st elution; E_2_: 2nd elution; L: total cell lysate; F: flow-through elution; (**B**) Protein score got from mass spectrometry. Protein score reflects the combined scores of all observed mass spectra that can be matched to amino acid sequences within that protein. A higher score indicates a more confident match. The number of protein matches at each scoring position is indicated by the height of the red bars. The non-significant area is shaded in green. Most matches are non-significant. However, one protein match is strongly significant; it is *B. mori* V-ATPase subunit H with protein score 129; (**C**) Summary of top ranking protein. The score value listed within the query table is the Ion Score. The Ion Score is a measure of how well the observed MS/MS spectrum matches to the stated peptide. The confidence threshold for Ion Scores is the same as that for the Protein Score. Ions Scores >28 were statistically significant (*p* < 0.05). The expected value indicates the probability that the observed match between MS/MS spectra and peptide sequence would be found by chance. Confident matches typically have expected values <0.1.

Using the antibody of His-tag, the western blotting result reveals that the purified His-tag protein has the same molecular weight of subunit H of *M. separata* (Figure 4A). Analyzed by LC-MS/MS, the result searched by the Mascot shows that the recombinant protein matched with the *B. mori* V-ATPase subunit H data (GenBank Accession No. ABF51492), the protein score is 129 (Figure 4B,C).

## 3. Discussion

*MsV1H* cloned from *M. separata* larvae is the first V-ATPase gene from this insect species. Based on the full length sequence, MsV1H was successfully expressed in insect sf9 cells and purified. These results may establish a foundation of further study on MsV1H crystal structure and function.

We investigated the developmental expression pattern of subunit H. The results show that the expression level reached the peak in sixth instar larvae and then dropped at pupal and adult stages. Moreover, no significant difference was observed between males and females at adult stage. As V-ATPases are located in endomembranes of insect cells and apical cell membrane of goblet cells of midgut, especially for Lepidoptera midgut, which is a rich source of V-ATPases, the expression pattern is consistent with the development of *M. separata*, The number of midgut cells increases and the larvae need more energy to survive with the development of *M. separata* larvae, so the expression level goes up from the first instar to sixth instar. However, for the pupal stage, they do not need lots of energy as they prepare for the differentiation of adult midguts, so the levels dropped. Compared with the larvae, the possible reason for the lower expression level at adult stage is that the pH of adult midgut is lower than larvae, as the main function of insect V-ATPase is to alkalize the gut lumen.

**Figure 5:**
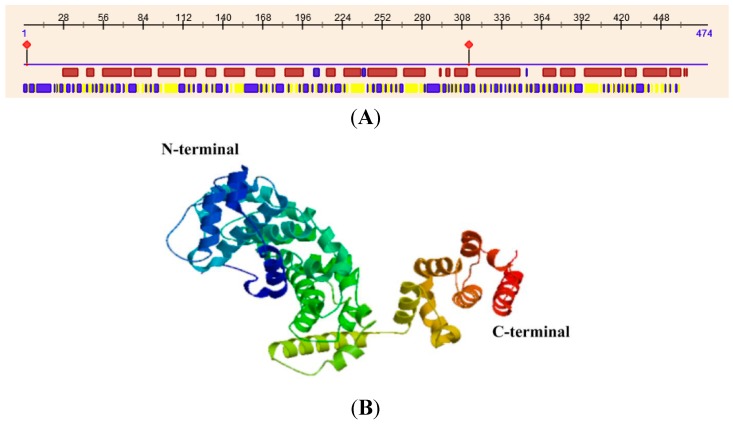
Secondary and tertiary structure of MsV1H. (**A**) Secondary structure of MsV1H. The first line shows the secondary structure of MsV1H from RePROF prediction methods. Red boxes indicate helix, while blue boxes indicate strand. The second line shows the residues that expose to the outside or buried inside of the protein. Blue boxes and yellow boxes show exposed and buried residues, respectively. The first and 310th residues show the predicted protein binding sites; (**B**) Tertiary structure of MsV1H produced by Swiss-Model.

The crystal structure of subunit H in yeast reveals that it is an elongated protein consisting of two major domains, the *N*-terminal and *C*-terminal domains. The *N*-terminal domain (residues 1–348), which consists of a series of five HEAT repeats, is joined with the *C*-terminal domain (residues 353–478) through a four-amino acid linker (residues 349–352). Hence, structural flexibility exists between the two domains [7,11]. The two domains make complementary contributions to structural and functional coupling of the peripheral V_1_ and membrane V_0_ sectors of the V-ATPase, but this coupling does not require that they be joined covalently. The *N*-terminal domain alone is enough to activate V_1_ complex to hydrolyze ATP, whereas the *C*-terminal domain is essential for proper communication between the V_1_ and V_0_ sectors [11]. Although MsV1H shares low amino acid identity (30%) with yeast, its secondary structure predicted by the web server PredictProtein (http://www.predictprotein.org/) and its tertiary structure produced by Swiss-Model is similar with yeast (Figure 5). The *N*- and *C*-terminal domains are primarily α-helices, from which we can deduce that the two terminals of MsV1H may have the same function as in yeast. Blastp result shows that 377–447 residues of *C*-terminal contain armadillo-like repeats. A single ARM repeat consists of ~42 residues that fold into three α-helices with an almost triangular cross section. Multiple ARM repeats pack regularly side by side, forming elongated molecules with a superhelical twist that results in an internal concave surface formed by the third helix of each repeat [12,13]. Proteins containing this domain interact with numerous other proteins. Through these interactions, they are involved in a wide variety of processes including carcinogenesis, control of cellular ageing and survival, regulation of circadian rhythm and lysosomal sorting of G protein-coupled receptors. In general, the structure of the H subunit decides its function.

V-ATPase Subunit H is essential for the catalysis but not for the assembly of the enzyme [11,12,14]. In a yeast vma13∆strain, which lacks the H subunit, V_1_-V_0_ complexes can still be assembled and transported to the vacuole, but this assembled whole enzyme is not active. When V_1_ and V_0_ complexes are disassembled, the H subunit remains with the V_1_ sector and acts as an inhibitor of ATP hydrolysis in the free V_1_ sectors [11]. Cysteine-mediated cross-linking results indicate that subunit H and subunit F are proximal in the free V_1_ domain but not in the intact V_1_V_0_, which supports the conclusion. Besides, the study indicates that the *C*-terminal domain of H subunit interacts with the B subunit in the dissociated V_1_ complex [7]. Furthermore, because the *N*-terminal domain is composed of a great many of α-helices and contains a hydrophobic groove which is similar to that in importins, subunit H is probably the binding site for other proteins which interact with V-ATPase. In fact, data of human V1H suggest the determination of a shared domain in β-adaptins and the regulatory subunit H of V-ATPase. The di-leucine-based internalization motif of Nef binds to the ARM repeat structure of V1H, which exhibits sequence homology to β-adaptins [12]. In addition, other studies show that the subunit H can interact with the adaptor protein complex 2 and the Golgi ectoapyrase [7]. All of these above indicate the important function of subunit H.

In recent years, an increasing number of studies indicate that the malfunction of V-ATPase is correlated with many human diseases, such as osteoporosis, male infertility or tumor metastasis [12,15,16,17,18]. So, it is quite necessary to reveal the mode of action of the enzyme and screen the novel inhibitors [8]. So far, a great many inhibitors of V-ATPase have been found. Referring to the review published by Markus Huss and Helmut Wieczorek [8], these inhibitors can be classified as three groups: the old players (the plecomacrolides bafilomycin and concanamycin), the new players (the macrolacton archazolid, the benzolactone enamides salicylihalamide, lobatamide, apicularen, oximidine and cruentaren, and the indolyls) and others [19,20,21,22,23]. Later, Chan *et al.* reported that alexidine dihydrochloride and thonzonium bromide, as a new class of inhibitors, uncoupled the proton transport and ATPase activity of the pump [24]. The binding sites of all these inhibitors are still elusive, even for the old players such as bafilomycin or concanamycin. Interestingly, so far, the results of all studies on the binding sites of some inhibitors just indicated that these inhibitors could interact with subunit c or V_0_ complex; none of them interacted with subunit H or V_1_ complex. Therefore, it is possible to design and screen the inhibitors to develop novel insecticide based on the structure of MsV1H.

In this study, cloning, expressing and purifying MsV1H in insect cells through Baculovirus Expression System is the first step of getting crystal MsV1H protein and clarifying its detailed structure for further screening of candidate activate compound. The western blotting and mass spectrometry results show that the recombinant protein has been successfully expressed and purified, and the Eukaryotic expression system has its advantages for expressing eukaryotic protein, but verification of the correctness of folding and its function needs to be done in the following work.

## 4. Experimental Section

### 4.1. Insects

Larvae of *M. separata* (Lepidopteran) were provided by our laboratory. The *M. separata* colony has been maintained in the laboratory for 17 years at 25 °C, 70% relative humidity, and a photoperiod of 16 h:8 h light:dark with periodic introduction of field-collected insects. 

Midguts were dissected from sixth instar larvae of *M. separata*. The whole midguts were removed and the contents were discarded. The tissue from 5 larvae was immediately frozen in liquid nitrogen.

### 4.2. Chemicals

RNA isolated reagent (RNAiso Plus), pMD20-T vector, 3'-RACE kit, 5'-RACE kit, PCR kit, and DNase I were purchased from TaKaRa Biotech. (Dalian, China). BacPAKTM Baculovirus Expression System Components was purchased from Clontech (Code No. 631413). Fungizone^®^ Antimycotic (liquid) was purchased from Invitrogen (Shanghai, China). Ni-NTA Spin column was purchased from GIAGEN (Shanghai, China). All other chemicals were purchased from Amresco (Solon, OH, USA) and Guanghua Sci-Tech (Guangdong, China).

### 4.3. Total RNA Isolation and cDNA Synthesis

The frozen midgut tissue was ground into a fine powder in liquid nitrogen and transferred into a centrifuge tube. One milliliter RNAiso Plus reagent was added and mixed, incubated at room temperature for 5 min, then centrifuged at 12,000× *g*, 4 °C for 5 min. After that, the supernatant was transferred into a new centrifuge tube, 200 μL of chloroform was added, and it was incubated at RT for 5 min. Later, the mixture centrifuged at 12,000× *g* (4 °C) for 15 min, after which the supernatant was transferred into a new centrifuge tube. Then, 500 μL isopropanol was added and it was centrifuged at 12,000× *g* (4 °C) for 10 min. After that, the pellet was washed with 75% ethanol. Finally, dried RNA was dissolved in DEPC-treated ddH_2_O and the concentration of RNA measured using a spectrophotometer (NanoDrop 200c, Thermo Scientific, Shanghai, China).

TaKaRa 3'-Full RACE Core Set Ver.2.0 (Code No. D314) kit was used to synthesize cDNA as outlined in the instructions, and at the same time, M-MLV (-) was set up as negative control. Total RNA (630 ng/μL) 1.6 μL, 3' RACE Adaptor (5 μM) 1 μL, dNTP mixture (10 mM each) 1 μL and RNase Free ddH_2_O 3.9 μL were added in a microcentrifuge tube, and the tube was later heated at 70 °C for 10 min, then immediately cooled on ice for 2 min. After briefly spinning, 5× M-MLV Buffer 2 μL, RNase Inhibitor (40 U/μL) 0.25 μL and Reverse Transcriptase M-MLV (RNase H-) (200 U/μL) 0.25 μL were added in the tube, mixed gently and incubated at 42 °C for 60 min, then heated at 70 °C for 15 min to inactivate Reverse Transcriptase. The product was used as PCR template. 

### 4.4. PCR and RACE

#### 4.4.1. PCR and RACE Primers

Three subunit H of V-ATPase amino acid sequences of several insects obtained from NCBI were compared with Clustal Omega (http://www.ebi.ac.uk/Tools/msa/clustalo/). The accession numbers are as follows: *Bombyx mori* DQ443403, *Manduca sexta* AJ249389.1, *Locusta migratoria* *manilensis* JQ244769.1. One pair of primers was designed according to the characteristics of the conservative region. These primers were used to amplify fragments of the V-ATPase subunit H gene of *M. separata.* Specific primers for RACE were derived from the sequence of the above PCR sequencing. The sequences of all primers used in this study are given in Table 1 and the relative positions of PCR and RACE primers are shown in Figure 1. All primers were synthesized by Invitrogen (Shanghai, China).

#### 4.4.2. PCR and RACE Reactions

PCR was carried out in a thermal cycler (SENSO, Beijing, China). The reaction mixture contained 8 μL× cDNA Dilution Buffer II, 2 μL 10 μM specific primer (or 3' RACE Primer or 5' RACE Primer ), 2 μL cDNA template, 2× GC Buffer I 25 μL, 0.5 μL TaKaRa LA Taq^®^ (5 U/μL) (TaKaRa, Dalian, China) and ddH_2_O 10.5 μL. PCR amplification was conducted using the following parameters: denaturation at 94 °C for 3 min; followed by 30 cycles of 94 °C for 30 s, 60 °C for 30 s and extension at 72 °C for 1 min; then a final extension at 72 °C for 10 min. The 5'-RACE and 3'-RACE kits reaction were conducted following the manufacturer’s protocol.

#### 4.4.3. Cloning and Identification of PCR and RACE Products

PCR and RACE products were separated by electrophoresis on 1% agarose gels in TAE buffer. The resulting band was visualized by ethidium bromide staining and the target fragment was cut and purified by TaKaRa Agarose Gel DNA Purification Kit Ver.2.0 (Code No. DV805A; Dalian, China. The purified target fragment was ligated with TaKaRa pMD20-T Vector (Code No. D107; Dalian, China) and transformed into *E. coli* Competent Cells JM109 by 90 s heat shock at 42 °C followed by growing for 45 min at 37 °C in 800 μL of LB medium. LB agar plates containing ampicillin were inoculated and incubated overnight at 37 °C, and the positive clones on the LB agar plates were identified by PCR amplification.

#### 4.4.4. DNA Sequencing and Analysis

Positive recombinants were selected and inoculated into liquid LB medium containing 100 μg/μL ampicillin. After an overnight incubation at 37 °C, plasmid DNA was extracted and sequenced.

#### 4.4.5. Sequence Analysis, Multiple Sequence Alignment, and Phylogenetic Analysis

Sequences of subunit H of V-ATPase obtained were analyzed using the BLAST algorithm at NCBI for comparative analysis. The deduced amino acid sequence of *MSV1H* was analyzed with the Expert Protein Analysis System (http://www.expasy.org/) and SMART program (http://smart.embl-heidelberg.de/). The signal peptide was predicted with SignalP 3.0 (http://www.cbs.dtu.dk/services/SignalP/). The potential sites of Asn-linked glycosylation were predicted with NetNGlyc 1.0 (http://www.cbs.dtu.dk/services/NetNGlyc). The phylogenetic tree was constructed using the neighbor-joining method in MAGE 5 software (Tempe, AZ, USA).

### 4.5. Real Time Quantitative PCR (qPCR)

To study stage specific expression of *MsV1H*, qPCR was carried out. *M. separata* individuals of different stages were used to isolate total RNA as described before, using three independent extracts. The first strand cDNA of each RNA sample for qPCR was synthesized using 3.0 μg of total RNA using RevertAid First Strand cDNA Synthesis Kit (Thermo Scientific, Shanghai, China) in a 20 μL reaction mixture as described by the manual. The relative expression levels of *MsV1H* at each developmental stage were assessed using the reference gene *M. separata* beta-actin (*MsActin*, GenBank number: GQ856238.1) as a reference. The primers used were QHF and QHR (Table 1) for *MsV1H* gene amplification, and QActinF and QActinR (Table 1) for endogenous control. The qPCR was performed on a BioRad CFX96 (BioRad, Hercules, CA, USA) using an UltraSYBR Mixture (CWBIO, Beijing, China) with 20 μL reaction mixture consisting of 10 μL UltraSYBR Mixture, 2 μL cDNA, 0.4 μL of each 10 μM forward and reverse primers, and 7.2 μL Nuclease-Free Water under the following cycling program: 1 cycle at 95 °C for 10 min, 40 cycles at 95 °C for 10 s, 55 °C for 30 s, 72 °C 30 s and a melting curve program (65–95 °C in increments of 0.5 °C, every 5 s) was conducted after PCR to confirm if the nonspecific product was produced. The standard curves of both *MsV1H* and *MsActin* were obtained using a linear gradient dilution (10-fold dilution) of cDNA as template. The relative *MsV1H* mRNA expression was calculated according to the 2^−ΔΔ*C*t^ method. A negative control (NTC) reaction was carried out using RNase-free water instead of cDNA templates. Each experiment contains three biological replicates, and each sample was technically repeated three times.

### 4.6. Expression and Purification of Subunit H

Based on the previous RACE result, the 1425 bp Coding sequence of *M. separata* was generated by PCR-mediated amplification with primer containing *EcoR* I*/Xho* I restriction sites, and 6× His tag was added before the stop codon TAA, then the sequence was sub-cloned to the Baculovirus Expression Vector, pBacPAK9 (Clontech, Dalian, China). Following the procedure recommended, P1 and P2 generation of baculovirus was obtained. Determined by PFU method, the recombinant baculovirus titer was 8 × 10^8^ ifu/mL. After adding the antibiotic (Fungizone^®^ Antimycotic, Invitrogen, Shanghai, China), the control and recombinant baculovirus were stored at 4 °C for further use.

Sf9 cells were infected with either control baculovirus or recombinant baculovirus encoding subunit H of *M. separata*. Ninety-six hours post-infection, cells were harvested by centrifugation (1000× *g* for 5 min) and the protein was purified by following the handbook of Ni-NTA columns (GIAGEN, Shanghai, China). Cells were washed once in PBS buffer (50 mM potassium phosphate; 150 mM NaCl, pH 7.2), re-pelleted, and resuspended in lysis buffer (50 mM NaH_2_PO_4_; 300 mM NaCl; 10 mM imidazole; 1% NP-40, pH 8.0). Incubating on ice for 30 min, lysate was centrifuged at 12,000× *g* for 30 min at 4 °C to collect the supernatant. After loading up to 600 μL of the cleared lysate containing the 6× His-tagged protein onto the pre-equilibrated Ni-NTA spin column, the column was centrifuged for 5 min at 270× *g*. The Ni-NTA spin column was washed twice with 600 μL Wash Buffer (50 mM NaH_2_PO_4_, 300 mM NaCl, 20 mM imidazole, pH 8.0) and centrifuged for 2 min at 890× *g*. Then, the recombinant protein remained on the column was eluted twice with 300 μL Elute Buffer (50 mM NaH_2_PO_4_, 300 mM NaCl, 500 mM imidazole, pH 8.0) and the column was centrifuged for 2 min at 890× *g* to collect the eluate. The eluate was dialyzed against 20 mM Tris pH 7.2 and lyophilized for identification.

### 4.7. Identification of Recombinant Protein by Western Blotting and Mass Spectrometry

Western blotting was carried out by dissolving the samples in 60 μL Tris-HCl (pH 7.2). After that, samples were electrophoretically separated on 12% SDS-PAGE gel and transferred onto nitro cellulose (NC) membranes using semi-dry Western blot transfer system (BioRad Inc., Hercules, CA, USA) for 30 min at 10 V. The membrane was then washed with PBST (PBS containing 0.02% Tween-20) and blocked with 5% non-fat dry milk for 2 h at room temperature (RT). Thereafter, membrane was washed three times in PBST (10 min each), and incubated with anti His-Tag mouse antibody (1:769, CWBIO, Beijing, China) at room temperature for 3.5 h. Subsequently, the membrane was incubated with the secondary antibody horse radish peroxidase (HRP) conjugated goat anti-mouse antibody (1:5000) for 60 min at RT. After washing four times with PBST, the membrane was stained by using DAB kit (TIANGEN, Beijing, China). The protein mass spectrometry was conducted in Beijing Protein Institute by using LC/Q-TOF-MS.

## 5. Conclusions

In summary, we isolated the first gene of subunit H of V-ATPase from *M. separata* midgut, determined the gene expression level in different development stages, and successfully purified the recombinant protein by expressing it in the Baculovirus Expression System. The deduced secondary and tertiary structures revealed the similarity of structure and function with yeast subunit H.
